# The invasive proteome of glioblastoma revealed by laser-capture microdissection

**DOI:** 10.1093/noajnl/vdz029

**Published:** 2019-09-28

**Authors:** Thomas Daubon, Joris Guyon, Anne-Aurélie Raymond, Benjamin Dartigues, Justine Rudewicz, Zakaria Ezzoukhry, Jean-William Dupuy, John M J Herbert, Frédéric Saltel, Rolf Bjerkvig, Macha Nikolski, Andreas Bikfalvi

**Affiliations:** 1 INSERM U1029, Pessac, France; 2 LAMC, University of Bordeaux, Bordeaux, France; 3 KG Jebsen Brain Tumour Research Center, University of Bergen, Bergen, Norway; 4 University Bordeaux, INSERM UMR1053, BaRITOn Bordeaux Research in Translational Oncology, Bordeaux, France; 5 Oncoprot, TBM Core US005 University of Bordeaux, France; 6 Bordeaux Bioinformatics Center, CBiB University of Bordeaux, France; 7 Plateforme Protéome, University of Bordeaux, Bordeaux, France; 8 Disease Gene Discovery Limited, London, UK; 9 NorLux Neuro-Oncology, Department of Biomedicine University of Bergen, Norway; 10 Oncology Department, Luxembourg Institute of Health 84, Val Fleuri, Luxembourg; 11 LaBRI, UMR5800 University of Bordeaux, Talence, France

**Keywords:** glioblastoma, intratumor heterogeneity, invasion, patient-derived xenograft, proteomics analysis

## Abstract

**Background:**

Glioblastomas are heterogeneous tumors composed of a necrotic and tumor core and an invasive periphery.

**Methods:**

Here, we performed a proteomics analysis of laser-capture micro-dissected glioblastoma core and invasive areas of patient-derived xenografts.

**Results:**

Bioinformatics analysis identified enriched proteins in central and invasive tumor areas. Novel markers of invasion were identified, the genes proteolipid protein 1 (PLP1) and Dynamin-1 (DNM1), which were subsequently validated in tumors and by functional assays.

**Conclusions:**

In summary, our results identify new networks and molecules that may play an important role in glioblastoma development and may constitute potential novel therapeutic targets.

**Importance of the Study**

Our study deals with the identification of molecular players and regulatory circuits in glioblastoma development. Since glioblastomas are heterogeneous tumors composed of a tumor core and an invasive periphery, we have chosen a proteomics approach which takes into account this regional heterogeneity. We used laser-capture microdissection and state-of-the-art proteomics analysis of core and invasive tumor areas to identify molecular players and signatures.

**Key Points**

- Proteomics analysis on central and invasive areas reveals molecular heterogeneity.
- Membrane trafficking, cytoskeleton, and metabolism pathways are particularly enriched.
- Identification of new players in glioblastoma development.

Glioblastomas (GBM) are the most common and aggressive tumors from the central nervous system. Patient survival rate, despite therapeutic improvements, is about 15 months after tumor detection.^1,2^ GBM derive mainly from astrocytes but may contain oligodendrocytic components as well. They are characterized by increase in vascular growth, but also by tortuous blood vessels, that are poorly perfused and thrombose, resulting in hypoxia and, in turn, necrosis.^3^ Furthermore, single or contro-lateral invasion is observed.^4^ The Stupp protocol, commonly used as first-line therapy, is based on large tumor mass resection, local irradiation, and temozolomide treatment. Tumor recurrence is a constant feature and observed after a few months at which point patients may undergo second line surgery and antiangiogenic therapy (bevacizumab).^5^ However, evasive resistance to antiangiogenic treatment is observed with tumor recurrence in most of the cases, mainly due to secondary tumors formed from invasive cells. We have recently performed RNA sequencing for both central and invasive areas and identified a gene regulatory network with high connectivity.^6^

In this study, we performed laser-capture microdissection of core and invasive areas from patient-derived GBM xenografts followed by high-throughput proteomic analysis. We identified novel protein signatures including proteolipid protein 1 (PLP1) and Dynamin-1 (DNM1) and validated them in additional tumor samples. Finally, a functional validation was carried out in vitro. Thus, our results report novel unexpected proteins that are involved in glioblastoma development and may constitute novel therapeutic targets for preventing invasion following surgery.

## Materials and Methods

### Ethical Issues

Male RAGγ2C^−/−^ mice were housed and treated in the animal facility of Bordeaux University (“Animalerie Mutualisée Bordeaux”). All animal procedures were performed as according to the institutional guidelines and approved by the local ethics committee (agreement number: 5522).

Patients gave their consent prior tissue analysis according to the clinical guidelines. Informed written consent was obtained from all subjects (Department of Neurosurgery, Humanitas, Milan according to Humanitas ethical committee regulations).

### Intracranial Tumor Xenografts

P3 spheroids were prepared 3 days before implantation by seeding 10^4^ P3 cells in neurobasal medium with 0.4% methylcellulose (Sigma) in a U-bottom 96 wells plate (Falcon). After PBS washing, five P3 spheroids were stereotactically implanted into the brains of randomly chosen three Ragγ2C^−/−^ mice (8–12 weeks old). P3 cells have been extensively characterized and have molecular profile of the mesenchymal subgroup (from male patient, age 64; chromosomal aberrations + [Chr 7, Chr19, 20q], −[1q42-q43, Chr9, Chr10, 20p] − [PIK3R, CDKN2A/B]^7^). Briefly, GBM spheroids (5 spheroids of 10^4^ cells per mouse) were implanted into the right cerebral hemisphere using a Hamilton syringe fitted with a needle (Hamilton, Bonaduz, Switzerland), following the procedure already described,^6^ which consisted in an injection at Bregma 0, 2 mm left, 3 mm deep.

### Laser-Capture Microdissection

Coronal brain sections (10 µm thickness) were made using a CM3050-S microtome (Leica, Wetzlar, Germany) from snap-frozen samples. The sections were then dehydrated in a series of pre-cooled ethanol baths (40 s in 95% and twice 40 s in 100%) and air-dried. Laser microdissection of samples was performed using a PALM MicroBeam microdissection system version 4.6 equipped with the P.A.L.M. RoboSoftware (P.A.L.M. Microlaser Technologies AG, Bernried, Germany). Laser power and duration were adjusted to optimize the capture efficiency. Microdissection was performed at 5× magnification. Three tumors were analyzed for each condition, and five caps were collected for each tumor type. Three replicates were generated on serial sections for each brain tumor.

### Sample Preparation for Mass Spectrometry

Microdissected tissues were first incubated in RIPA buffer supplemented with inhibitor cocktail (Complete, Roche). They were then treated by sonication for 10 s, supplemented with Laemmli buffer, heated at 95°C during 5 min and finally loaded onto a 10% acrylamide SDS-PAGE gel. Migration was stopped when samples entered into the first centimeter of the resolving gel and proteins were visualized by Colloidal Blue staining. Each SDS-PAGE band was cut into 1 × 1 mm gel pieces. Gel pieces were destained in 25 mM ammonium bicarbonate (NH_4_HCO_3_), 50% Acetonitrile (ACN), and shrunk in ACN for 10 min. After ACN removal by evaporation or pipetting, gel pieces were dried at room temperature. Proteins were first reduced in 10 mM dithiothreitol, 100 mM NH_4_HCO_3_ for 30 min at 56°C, then alkylated in 100 mM iodoacetamide, 100 mM NH_4_HCO_3_ for 30 min at room temperature, and shrunken in ACN for 10 min. After ACN removal, gel pieces were rehydrated with 100 mM NH_4_HCO_3_ for 10 min at room temperature. Before protein digestion, gel pieces were shrunken in ACN for 10 min and then dried at room temperature. Proteins were digested by incubating each gel slice with 10 ng/µl of trypsin (T6567, Sigma–Aldrich) in 40 mM NH_4_HCO_3_, 10% ACN, rehydrated at 4°C for 10 min, and finally incubated overnight at 37°C. The resulting peptides were extracted from the gel by three steps: a first incubation in 40 mM NH_4_HCO_3_, 10% ACN for 15 min at room temperature and two incubations in 47.5% ACN, 5% formic acid for 15 min at room temperature. The three collected extractions were pooled with the initial digestion supernatant, dried in a SpeedVac, and resuspended in 0.1% formic acid before nanoLC-MS/MS analysis.

### nanoLC-MS/MS Analysis

Online nanoLC-MS/MS analyses were performed using an Ultimate 3000 RSLC Nano-UPHLC system (Thermo Scientific, USA) coupled to a nanospray Q-Exactive hybrid quadruplole-Orbitrap mass spectrometer (Thermo Scientific, USA). Each peptide extract were loaded onto a 300 µm ID × 5 mm PepMap C_18_ precolumn (Thermo Scientific, USA) at a flow rate of 20 µl/min. After 3 min desalting, peptides were online separated on a 75 µm ID × 25 cm C_18_ Acclaim PepMap RSLC column (Thermo Scientific, USA) with a 4–40% linear gradient of solvent B (0.1% formic acid in 80% ACN, solvent A: 0.1% formic acid in H_2_O) in 108 min. The separation flow rate was set at 300 nl/min. The mass spectrometer was operated in positive ion mode at a 1.8 kV needle voltage. Data were acquired using Xcalibur 3.1 software in a data-dependent mode. Full MS scans in the range from m/z 300 to 1600 were recorded at a resolution of 70,000 at m/z 200 and the target value for the automatic gain control (AGC) algorithm was set to 3 × 10^6^ ions collected within 100 ms. Dynamic exclusion was set to 30 s and top 12 ions were selected from fragmentation in HCD mode. MS/MS spectra were acquired with a resolution of 17,500 at m/z 200, and the maximum ion injection time and the AGC target were set to 100 ms and 1 × 10^5^ ions, respectively. Only precursors with assigned charges states +2 and +3 were selected for fragmentation. Molecules that are not proteins are generally charged with only one proton, hence the interest of sequencing only multicharged ions, which are generally peptides. Furthermore, peptide anion analysis remains little practiced because of challenges with high-pH reversed-phase separations and a lack of robust fragmentation methods suitable for peptide anion characterization. Others settings were as follows: no sheath and no auxiliary gas flow; heated capillary temperature of 270°C; normalized HCD collision energy of 27%; and an isolation width of 2 m/z.

### MS Data Processing and Quantification

Mascot 2.5 algorithm through Proteome Discoverer 1.4 Software (Thermo Fisher Scientific Inc.) was used for protein identification in batch mode by searching against a *Mus musculus* database (Proteome ID UP000000589; release date November 15, 2018; 54,188 proteins) merged to a *Homo sapiens* database (Proteome ID UP000005640; release date November 17, 201pretty8; 73,931 proteins) from http://www.uniprot.org/ website.^8^ Two missed enzyme cleavages were allowed. Mass tolerances in MS and MS/MS were set to 10 ppm and 0.02 Da. Oxidation of methionine, acetylation of lysine, and deamidation of asparagine and glutamine were searched as dynamic modifications. Carbamidomethylation on cysteine was searched for as a static modification. For protein quantification, raw LC-MS/MS data were imported in Proline Studio (http://proline.profiproteomics.fr/) for feature detection, alignment, and quantification. Protein identification was only accepted when at least 2 specific peptides had a pretty rank = 1 and had a protein false discovery rate value < 1.0% calculated using the “decoy” option in Mascot.^8^ Label-free quantification of MS1 level by extracted ion chromatograms was carried out using the parameters indicated in Supplementary Table 1. Protein ratios were normalized to the median ratio. A missing values inference method was applied, and we used a variant when there is a minimum of 1.5-fold change with an adjusted *P* value below .05.

### Experimental Reproducibility Between Triplicates

Protein quantifications were analyzed using a bespoke pipeline of python scripts. For human data set, there were 743 proteins in each brain sample and 517 proteins for the mouse data set. We estimated the dispersion of protein quantification values between technical replicates (3 per sample) for each condition (angiogenic and invasive) by computing the coefficient of variation CV= σμ, where σ and μ are the standard deviation and mean. Histograms of CV values were built for each sample separately for each condition. Based on the analysis of these histograms, we chose CV=0.8 as the threshold for eliminating proteins whose values were not sufficiently reproducible between triplicates. Proteins were retained only if the CV was below the 0.8 threshold for both conditions, resulting in 574, 691, and 714 proteins for each of the brain samples for Human and in 372, 468, and 484 proteins for each of the brain samples for mouse. To compare central and invasive conditions, we have generated aggregated tables with protein quantifications for all samples and replicates, resulting in 18 column tables (9 columns per condition) for Human and mouse; a protein was retained to be part of these tables only if it was present in 6 out of 9 columns for each condition. These resulting aggregated data set contain in total 730 proteins for the Human-annotated data set and 510 for the mouse-annotated data set (Supplementary Tables 2 and 3). Furthermore, for certain downstream analyses, we considered only proteins common to all replicates, reducing the aggregated data sets to 544 and 331 proteins for Human and mouse, respectively.

### Differential Expression Analysis

To reveal potential biomarkers which distinguish between invasive and angiogenic conditions, we applied the Welch’s *t*-test to the corresponding replicate value vectors. The resulting *P*-values were further adjusted using the Benjamini–Hochberg multiple test correction algorithm^9^ resulting in padj values for each protein. We set the threshold for the significance of the padj at 0.01, yielding (i) for the aggregated data sets 152 and 284 potential biomarkers for Human and mouse, respectively; and (ii) for the common data sets 119 and 206 potential biomarkers for Human and mouse, respectively. These significantly differentially expressed proteins are further filtered by computing the log-fold change between protein quantifications for invasive and angiogenic replicates as $\log FC=\log{}^{\mu (I)}╱{}_{\mu (A)}$, where I and A are vectors of values for invasive and angiogenic conditions, respectively. Proteins having padj<0.01 and |logFC|>2 for the Human data set and padj<0.01 and |logFC|>5 for the mouse data set are retained to generate the clustermaps. Clustermaps were generated using pheatmap package v.1.0.12^10^ with the Euclidean distance and Ward D2 clustering method parameters.

### Functional Enrichment Analysis

For the enrichment analysis, the significantly differentially expressed proteins (padj<0.01) in the aggregated data sets were further filtered by the logFC criterion. We have retained proteins having the |logFC|>1 for both Human- and mouse-aggregated data sets. In order to find pathways deregulated between the two conditions of interest, we subsequently performed a Gene Set Enrichment Analysis (GSEA) on these subsets. We used Gseapy python package (https://pypi.python.org/pypi/gseapy), which is a wrapper to the functionalities provided by the Broad’s institute GSEA suite (http://www.broad.mit.edu/gsea/). We used the enrichment function, applying a 0.05 adjusted P-value cutoff, and ran the analysis using the GO biological process and GO cellular component knowledgebases. KEGG package was also used for defining cellular pathways enriched in invasive areas.

### AngioScore

The AngioScore was calculated as done previously.^11^ Briefly, for each gene, the AngioScore is the percentage of publications that contain one or more relevant angiogenesis keywords in the abstracts from all publications assigned to a gene by the Entrez Gene database. A *t*-test comparison of AngioScores between the core and the invasive GBM areas was performed.

### Antibodies and Reagents

PLP inhibitor peptide was purchased from Peprotech (100–21), and DNM1 inhibitor, Dynasore (RD Systems, 201-LB/C). Cells were treated at concentrations indicated concentrations in the legend section.

### Histological and Immunohistological Analyses

For immunofluorescence on histological sections, frozen sections were processed as described previously.^6^ Patient paraffin-embedded sections were deparaffinised in xylene and hydrated serially in 100%, 95%, and 80% ethanol. Endogenous peroxidase was quenched in 3% H2O2 in PBS for 10 min. Slides were then incubated with anti-DNM1 (Invitrogen PA1660), anti-PLP1 (Sigma SAB2101830), or anti-Nestin (ThermoFisher PA5-11887) antibodies overnight at 4°C. Sections were washed three times in PBS, and secondary fluorescent antibodies were applied (Anti-mouse or anti-rabbit fluorescent antibodies). After mounting, DNM1 or PLP1 expression localizations were analyzed using IHC profiler from Fiji Software. IHC profiler uses the DAB signal in images and the results are expressed as a ratio the DAB area to the total area.

### Proliferation and Viability Experiments

A 96 wells plate (Falcon) was coated with 0.2 mg/ml of Matrigel in NBM for 30 min. Then, 2000 P3 cells were placed into the wells with 10 ng/ml of Hoechst stain in NBM and incubated for 24 h. When adherent cells appeared, supernatant were removed and substituted by 100 µl of NBM with treatment. Pictures of each well were taken at T0, 24 h, 48 h, and at 72 h. LIVE/DEAD (Invitrogen) assays were also realized through the addition of Calcein into the wells. The number of living (in blue) and/or dead (in red) cells was quantified with the FIJI software.

### Invasion Assays in Collagen I Gels

P3 spheroids were prepared 3 days, respectively, before inclusion by the seeding of 10^4^ cells in neurobasal medium with 0.4% methylcellulose (Sigma) in a U-bottom 96 wells plate (Falcon). A solution of 1 mg/ml of collagen I (Fisher Scientific) was prepared in PBS with 7.2mM NaOH. Treatments were mixed directly into the collagen gels. After 30 min incubation on ice, spheroids were individually picked, washed in PBS, and included in the collagen solution. After 45 min at 37°C in a cell incubator, neurobasal medium with the different treatments was added. P3 spheroid invasion areas were measured after 24 h with FiJi software, with a home-made macro. Briefly, total area was automatically quantified and the core area was manually measured. The core area was then subtracted from the total area for obtaining the invasive area. For each independent experiment, the mean of 7 to 8 invasive areas was calculated and compared with controls.

### Statistical Analysis for In Vitro Experiments

Statistical analysis was performed using the Graphpad software. Multiple comparisons were performed with one-way analysis of variance, followed by Tukey post hoc tests and with one-way ANOVA Bonferroni multiple comparison test. Statistical comparison between two groups was performed by using the Mann–Withney test.

### Data Availability

The mass spectrometry proteomics data have been deposited to the ProteomeXchange Consortium via the PRIDE (Deutsch et al., 2017) partner repository, with the data set identifier PXD012791. All data are available within the Article and Supplementary Files, or available from the authors upon request.

## Results

### Laser Capture and Mass Spectrometry of Glioblastoma Core and Invasive Areas

**Figure 1.**
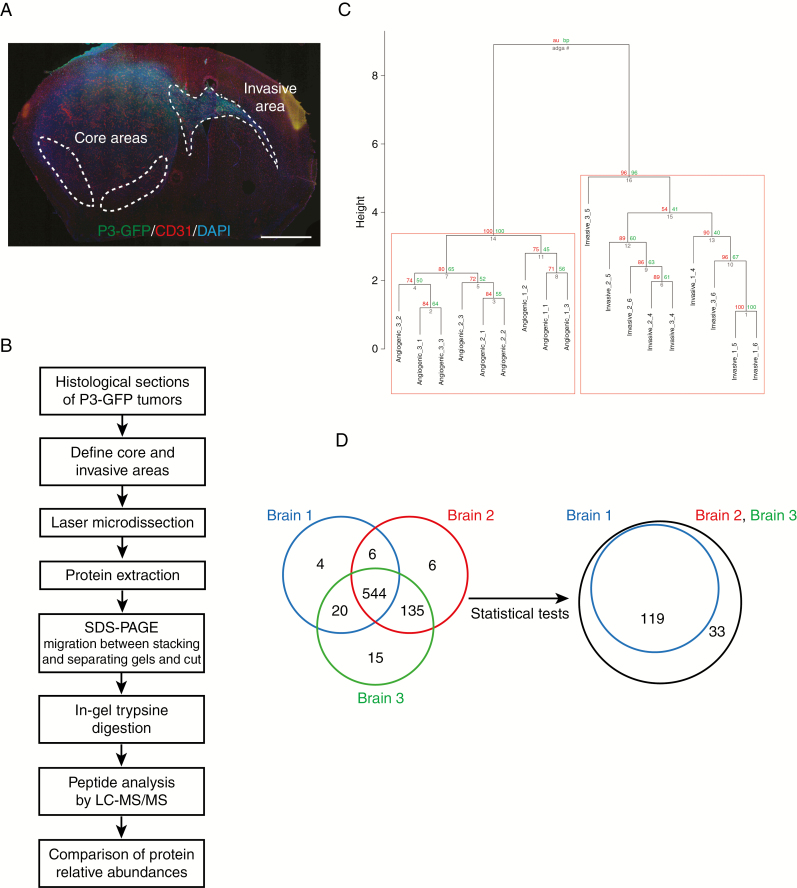
Laser-capture microdissection and proteomics analysis for comparing central and invasive glioblastoma areas. (A) Schematic representation of P3 tumor core and invasive areas. P3-GFP cells were stereotactically injected into mouse hemisphere and were visualized using fluorescence microscopy with CD31 and DAPI staining (P3 in green, CD31 in red, and DAPI in blue). Scale bar = 500 µm. (B) Technical ﬂow chart of P3 tumors analysis by combining laser microdissection and mass spectrometry analysis. (C) Hierarchical clustering of paired samples obtained from the analysis of the log-ratio values of the 544 proteins common for all samples. Values at the left represent approximately unbiased *P* values (AU) and values at the right correspond to boot- strap probability (BP). (D) Venn diagrams of Human proteins expressed in the three different tumors analyzed by proteomics. 544 proteins are common between the three tumors (left diagram). After Welch test *P*-values by FDR and setting the significance threshold at 0.01, 152 Human proteins were found differentially regulated between the core and the invasive areas (right diagram).

**Figure 2.**
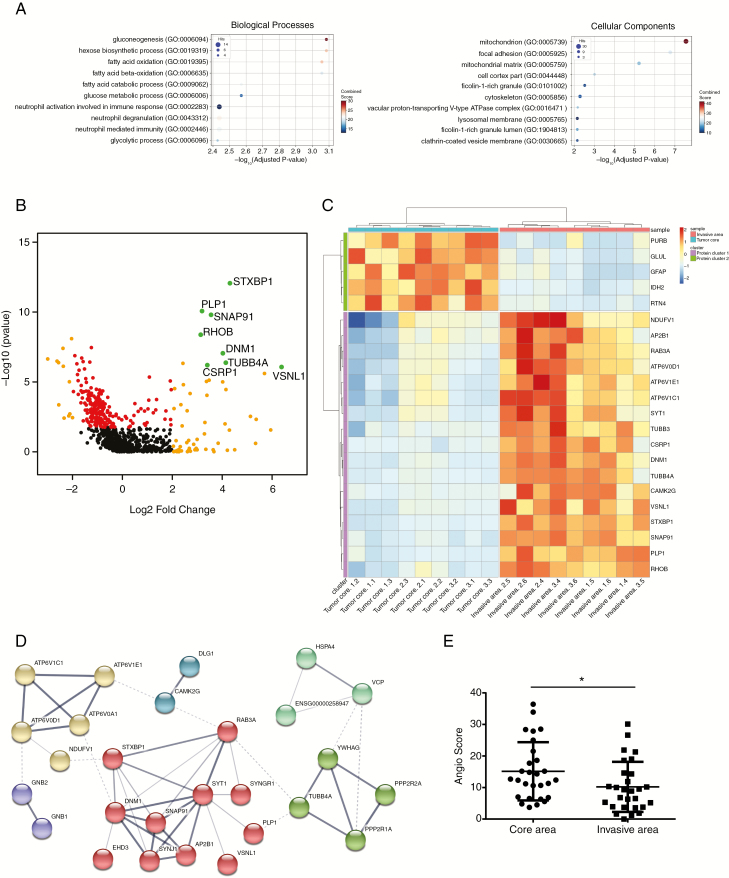
Protein enrichment of tumor invasive area (A) Gene Set Enrichment Analysis results. Gene Ontology (GO) biological processes (BP) and cellular components (CC) enrichment of the top 152 proteins that are signiﬁcantly differentially expressed between core and invasive areas (*p*val adj < 0.01 and |logFC| > 2), and which are represented here for the three tumors. The x-axis represents the negative log10 *P*-adjusted value. The size of each spot (Ratio) corresponds to the fraction of proteins within our set of proteins that have the corresponding GO function. (B) Graphical representation of quantitative proteomics data for the three P3 tumors. Proteins are ranked in a volcano plot according to *P* value for technical reproducibility, calculated from a one-tailed paired *t-*test (−log10 [P value]) (y-axis) and their relative abundance ratio (log ratio) between core and invasive areas (x-axis). The line indicates the *P* < 0.05 threshold. Off-centered spots are those that vary the most between core and invasive areas. (C) Heatmap of the top 22 of the 152 proteins that are signiﬁcantly differentially expressed for P3 tumor core and invasive areas (*p*val adj < 0.01 and |logFC| > 2). (D) Network of human tumor invasive proteins. Each node represents a protein and interactions with medium confidence >0.4 are showed. Proteins are clustered using the Markov Cluster Algorithm and colors represent clusters. Dashed lines represent intercluster edges and width represent edge confidence (medium: >0.4, high: >0.7, and highest: 0.9). Disconnected proteins are removed from the analysis. (E) AngioScore of the top 30 hits from P3 tumor core and invasive areas. Student *t*-test, * *P* < .05.

For this study, we have used patient-derived P3 tumors. These tumors are of mesenchymal phenotype and have the following characteristics: male patient, age 64; chromosomal aberrations + [Chr 7, Chr19, 20q], − [1q42-q43, Chr9, Chr10, 20p] − [PIK3R, CDKN2A/B]. Five P3 spheroids were injected in the right hemisphere in the striatum.^6^ Laser-capture microdissection (LCM) was performed on patient-derived glioblastoma xenografts from P3 cells transduced with GFP vectors from core and invasive areas and the proteins extracted (Figure 1A and B). Tumor core and invasive areas from three different mice were laser-microdissected and processed for proteomic analysis. Proteins were digested and analyzed by liquid chromatography/tandem mass spectrometry (LC-MS/MS). Relative abundances of specific Human and mouse peptides were compared and then grouped to analyze the differential protein expression levels. The dendrogram showed a distinct pattern of arborescence for invasive and core proteins (Figure 1C and Supplementary Figure 1A). A total of 730 Human and 510 mouse proteins were detected. A Venn diagram shows a comparison of the number of proteins found for three different mouse brains, where 544 Human proteins were found common with all (Figure 1D). After adjusting the Welch test *P*-values by FDR and setting the significance threshold at 0.01, 152 Human proteins were found significantly differentially regulated between the core and the invasive areas (Figure 1D), and 284 mouse proteins (Supplementary Figure 1B). Thirty-four Human proteins were found upregulated in the invasive area and 118 in core area (Supplementary Table 4). For Human proteins, metabolic pathways were enriched, notably gluconeogenesis (GO:0006094), mitochondrion (GO:0005739), and fatty acid metabolism (GO:019395 and GO: 0006635) (Figure 2A). Furthermore, categories relating to cytoskeleton proteins (focal adhesion GO:0005925, cell cortex part GO:0044448, and or cytoskeleton GO:0005856) and trafficking were also enriched (Figure 2A). Two hundred seventy-six mouse proteins were found upregulated in the invasive area and eight in core area (Supplementary Table 5). For mouse proteins, metabolic pathways were also enriched, such as gluconeogenesis (GO:0006094), glucose catabolic to pyruvate (GO:0061718), canonical glycolysis (GO:0061621), and mitochondrion (GO:0005739) (Supplementary Figures 1C and 2). Analysing invasive and central protein hits with KEGG database, PI3K-Akt signaling pathway (hsa04151), and synaptic vesicle cycle (hsa04721) were highly represented (Supplementary Table 6).

### Enrichment of New Marker Proteins in Invasive Glioblastoma Area

For the subsequent analyses, we focused our attention on the Human specific proteins. Volcano plot for proteins overexpressed in the invasive part and/or the tumor core demonstrated a clear enrichment of proteins in the core area, but with higher log-fold change (|logFC|) found in the invasive area (Figure 2B and Supplementary Table 4). A 2D cluster heatmap of proteins with padj<0.01 and logFC>2 and which are in common in three mouse brains showed a highly significant overexpression of Proteolipid Protein-1 (PLP1, log-fold change of 3.19), Dynamin-1 (DNM1, log-fold change of 4.02), and RHOB (log-fold change of 3.14) in the invasive area (Figure 2C). Among the most upregulated proteins in the core area were Glial Fibrillary Astrocytic Protein (GFAP, log-fold change of −2.38), Isocitrate Dehydrogenase 2 (IDH2, log2 fold change of −2.04), and glutamine synthetase (GLUL, log-fold change of −2.99) (Figure 2C). An interaction network of proteins from the invasive area showed a main node where DNM1 and PLP1 were linked (Figure 2D). Among the highest upregulated genes, VSNL1 (Visinin Like 1, log-fold change of 6.38) and STXBP1 (syntaxin binding protein, log-fold change of 4.31) were identified. However, functional analysis of these molecules is difficult since no specific inhibitors exist.

To provide further evidence of the difference between the invasive area and the angiogenic tumor core, we calculated an “AngioScore,” as described previously.^11^ The AngioScore is derived by searching angiogenesis and tumor angiogenesis keywords against total publications using Pubmed and reflects in our analysis angiogenic signaling provided by the tumor (Figure 2E). The average AngioScore was 1.5 times higher in the angiogenic tumor core when compared with the invasive area, by taking the 30 best hits from each area. This is globally in agreement with the proteomic expression analysis. By filtering the 20 highest AngioScores, most of the identified protein potential biomarkers were expressed in the core area (Supplementary Table 7). In the invasive part, some proteins (RHOB or fumarate hydratase) also exhibited an elevated AngioScore but these proteins have been indeed extensively studied in the context of tumor angiogenesis (Supplementary Table 7).

### Analysis of PLP1 and DNM1 Expression in Patient Samples

**Figure 3.**
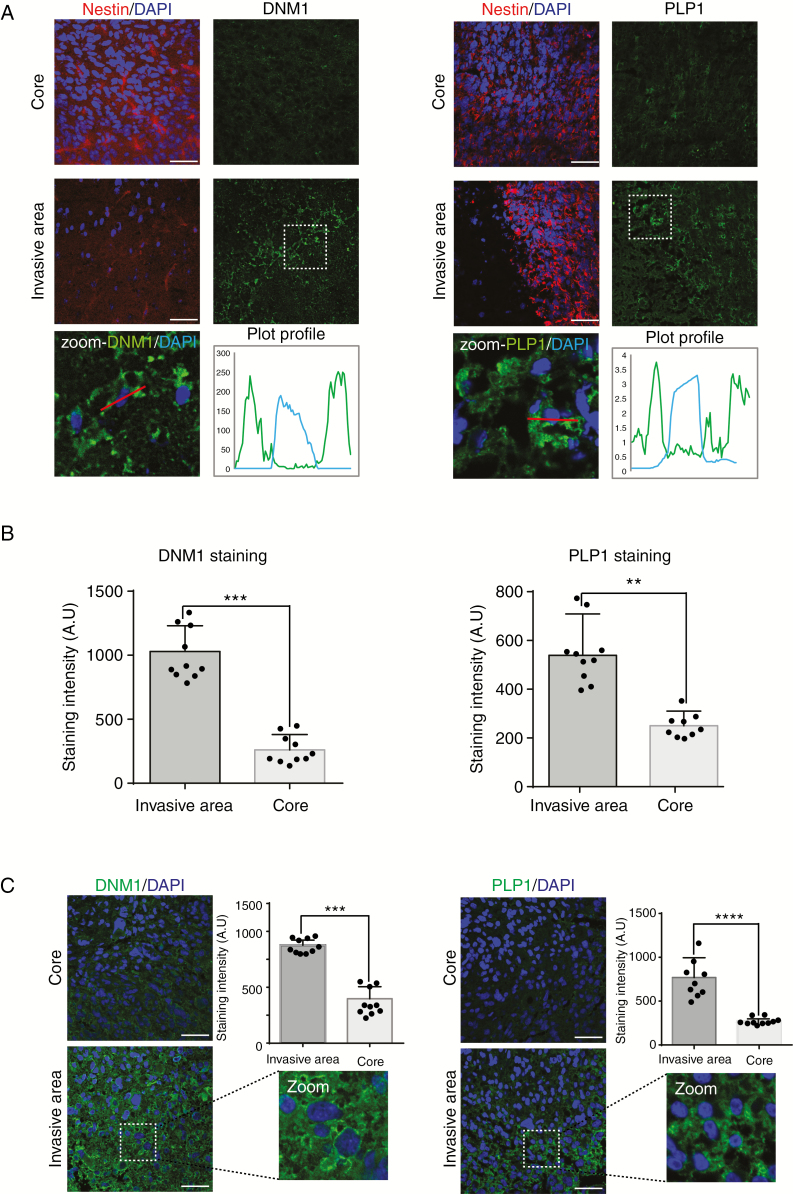
DNM1 and PLP1 are more expressed in invasive areas of patient-derived xenografts and in patients. (A) Immunostaining of Nestin (red) and DNM1 or PLP1 (green) in the tumor core (upper panels) and in the invasive area (middle panels) of P3 tumors. DAPI was used for nuclear staining (blue). Scale bars: 100 µm. Lower panels represent magnified images as delineated by dashed line squares, with plot profiles defined using Fiji software (defined by white lines). Both DNM1 and PLP1 are cytoplasmic. (B) The graphs represent DNM1 and PLP1 staining intensity of 10 different images from P3 core and invasive areas (Student *t*-test, ** *P* < .01; ***P < .001). (C) Immunostaining of DNM1 or PLP1 (green) in the tumor core (upper panels) and in the invasive area (lower panels) of P3 tumors. DAPI was used for nuclear staining (blue). Scale bars: 100 µm. The graphs represent DNM1 and PLP1 staining intensity of 10 different images of core and invasive areas from several patient sections (Student *t*-test, ***P < .001; ****P < .0001).

Little is known about the role of PLP1 in tumor development^12^ and only few publications identify DNM1 as glioblastoma marker.^13,14^ Therefore, we focused our attention on these proteins since they were significantly overexpressed in the invasive area. Immunostaining with anti-PLP1 or anti-DNM1 antibodies showed higher expression of both proteins in invasive areas in patient-derived xenografts (Figure 3A and B). PLP1 was detected in the extracellular space and on the plasma membrane, and DNM1 was expressed in the cytoplasm (Figure 3A and B). This was confirmed in paraffin-embedded patient samples (Figure 3C).

### PLP1 and DNM1 Inhibition Decreases Cell Invasion

**Figure 4.**
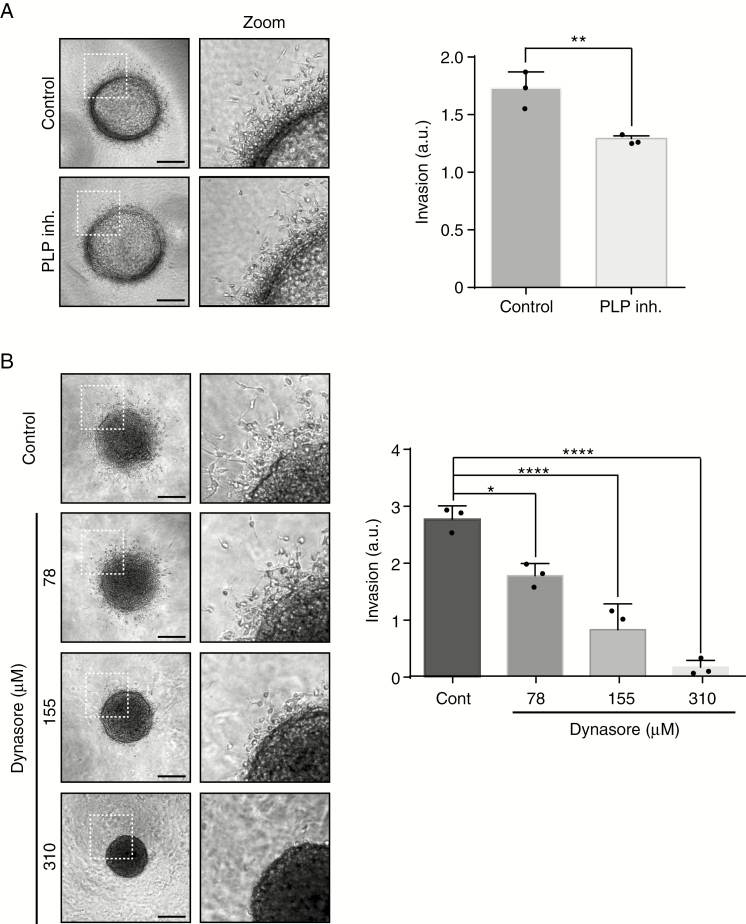
DNM1 and PLP1 control P3 cell invasion. (A) P3 cells were included into Collagen I gels and then incubated with control or PLP1 inhibitor (80 µg/ml). P3 spheroid invasion was measured in collagen I gels after 24 h. Scale: 50 µm. The graph represents the results as means ± SD of three independent experiments, each done in eight replicates for each condition. **P < .01 (Student *t*-test). (B) P3 cells were included into Collagen I gels and then incubated with control or DNM1 inhibitor Dynasore at several concentrations (78, 155, or 310 µM). P3 spheroid invasion was measured in collagen I gels after 24 h. Scale: 50 µm. The graph represents the results as means ± SD of three independent experiments, each done in eight replicates for each condition. *P < .05; ****P < .0001 (ANOVA).

**Figure 5.**
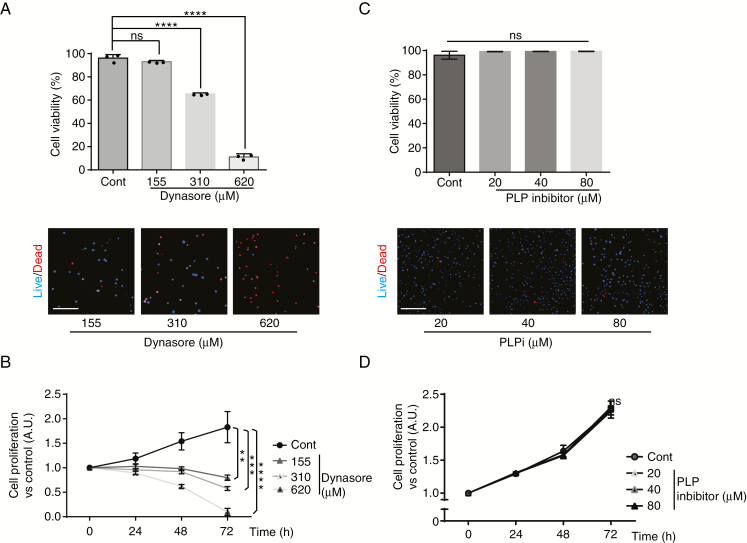
DNM1 inhibitor Dynasore reduces cell viability and proliferation at high doses. (A) Cell viability was recorded in live microscopy with Dead/Live kit (Invitrogen) as represented in lower panels (blue for living cells and red for dead cells). Dynasore was used at 155, 310, or 620 µM as indicated. The graph represents the percentage of living cells at the time of 72 h, and the results as means ± SD of three independent experiments, each done in 10 replicates for each condition. ns, nonsignificant; ****P < .0001 (ANOVA). (B) Cell proliferation was recorded in live microscopy at time 24, 48, and 72 h. Dynasore was used at 155, 310, or 620 µM as indicated. The graph represents cell proliferation at different time points, and the results as means ± SD of three independent experiments, each done in 10 replicates for each condition. ns, nonsignificant; **P < .01; ***P < .001; ****P < .0001 (ANOVA). (C) Cell viability was recorded in live microscopy with Dead/Live kit (Invitrogen) as represented in lower panels (blue for living cells and red for dead cells). PLP1 inhibitor (PLPi) was used at 20, 40, or 80 µM as indicated. The graph represents the percentage of living cells at the time of 72 h, and the results as means ± SD of three independent experiments, each done in 10 replicates for each condition. ns, nonsignificant (ANOVA). (D) Cell proliferation was recorded in live microscopy at time 24, 48, and 72 h. PLP1 inhibitor (PLPi) was used at 20, 40, or 80 µM as indicated. The graph represents cell proliferation at different time points, and the results as means ± SD of three independent experiments, each done in 10 replicates for each condition. ns, nonsignificant (ANOVA).

To functionally study the role of PLP1 and DNM1, invasion experiments were performed. We used the patient-derived cell line P3 for these experiments. To interfere with PLP1- and DNM1-dependent invasion, specific inhibitors were used in a collagen type I invasion assay (Figure 4). Both inhibitors showed inhibitory activity on invasion (29% of inhibition for PLP1 inhibitor; 36%/71%/97% of inhibition for Dynasore at 78/155/310 µM, respectively) (Figure 4A and B). The specific inhibitor of DNM1 (Dynasore) demonstrated a very strong and dose-dependent inhibition of tumor cell invasion (Figure 4B). To rule out cell toxicity, we used the Dead/Live kit assay. At a maximum inhibitory Dynasore concentration of cell invasion (310 µM), cytotoxicity was indeed observed but not at 155 µM, which inhibited invasion at 71% (Figure 5A). In addition, Dynasore at 155 µM also inhibited proliferation of P3 cells (Figure 5B). We also verified the effect of PLP inhibitor on cytotoxicity and proliferation. No difference in comparison to untreated cells was found (Figure 5C and D).

## Discussion

In this article, we undertook a systematic study to unravel molecular signatures related to glioblastoma development and, in particular, invasion. To this aim, we performed a proteomics analysis on patient-derived tumors implanted in immunodeficient mice. It has been previously shown that the molecular characteristics of patient tumors remain stable when xenografted in mice.^15^ We performed our analysis on laser-capture microdissected material obtained from invasive and central tumor areas. The scientific pipeline used in this study was validated in previous publications in which unidentified protein candidates were discovered for tumor classification^16^ or for invadopodia structure.^17^ When stringent statistical conditions were applied (padj<0.01 and logFC>2), 34 Human proteins were overexpressed and 118 were downregulated in invasive areas (out of 152 proteins present in the aggregated dataset).

Our analysis also included the estimation of an AngioScore. This latter approach was already successfully used in a previous publication from our team.^11^ In the AngioScore, we compared the 30 best hits between core and invasive areas, and confirmed enrichment angiogenesis-related proteins in the core area. Furthermore, the AngioScore was significantly reduced in the invasive area.

To reinforce the validity of our analysis, we only selected proteins which were overexpressed in the different tumor areas of three different animals. PLP1 and DNM1 were among the top candidates. PLP1 is a proteolipid protein expressed in the myelinated neurons, which was recently found in glioblastoma by scRNAseq.^18^ However, this article did not discriminate between the viable core and the invasive areas. PLP was shown to be expressed in glioblastoma without isoform distinction^12^ but more recently, the PLP1 isoform was reported as oligodendrocytic marker.^19^ We reinforced our analysis by immunohistology using a specific anti-PLP1 antibody, to ascertain its overexpression in the invasive area. Furthermore, we performed functional analysis using a specific PLP inhibitor and found that tumor cell invasion was inhibited. A similar analysis was performed on DNM1. DNM1 has been studied in acute myeloid leukaemia, lung, and colon adenocarcinomas but not in glioblastoma.^20^ In glioblastoma, DNM1 was recently characterized as a marker of long-term patient survival in a computational analysis.^21^ At a biological level, DNM1 was found to form a complex with Cortactin in glioma cells.^14^ Our data are not in agreement with a publication which reported a role of DNM2 in glioblastoma invasion using the standard glioblastoma cell lines LN444 and SNB19.^22^ DNM2 is highly expressed in all cancer types contrary to the expression of DNM1, which is mainly expressed in glioma, as referenced in TCGA. Furthermore, DNM2 is not a good prognosis marker for glioblastoma patient survival (TCGA database). Dynamin 2 was also recently described as a potential therapeutic target for glioblastoma development.^23^ In our analysis, 2′,3′-cyclic-nucleotide 3′-phosphodiesterase (CNP, log2 fold change of 1.28) was significantly overexpressed in the core area in agreement with a previously published study.^24^ CNP is not well studied in glioblastoma development and can be of interest for further studies. Furthermore, as indicated by the enrichment analysis, metabolic pathways were highly represented in both Human and mouse protein data sets.

We validated the regional expression of PLP1 and DNM1 by analyzing their RNA expression in the Ivy Glioblastoma Atlas Project database. Both candidates were found upregulated in the infiltrative tumor (IT), and the leading edge (LE) when compared with core areas (Cellular Tumor—CT). These areas were defined by histological characteristics, by evaluating the number of tumor cells in the sections. This correlation indicates that both tumor genes and proteins are involved in glioblastoma invasion.

Our study differs from the previous proteomics analysis where two different PDX glioblastoma models (angiogenic or invasive) were compared.^25^ In the latter analysis, Annexin A2 (ANXA2) and Calnexin (CNX) were found upregulated in the angiogenic model. A second proteomics study, from the same group, investigated effects of bevacizumab, which significantly modulated the tumor proteome.^26^ Bevacizumab induces a switch from angiogenic to invasive tumors, largely documented in the literature.^5,27^ Our study is completely different from these studies above since they did not take into account regional heterogeneity. Thus, the results from the proteomics cannot be compared, except for Isocitrate dehydrogenases 1 and 2 (IDH1, IDH2), ANXA5, and alpha-Enolase (ENO1) which were found upregulated in the angiogenic tumor model^26^ and in the core area in our study. All together, we highlight in this study underexplored proteins, which show evidence of a role in cell invasion.

## Supplementary Material

vdz029_suppl_Supplementary_Figure_1

vdz029_suppl_Supplementary_Figure_2

vdz029_suppl_Supplementary_Table_1

vdz029_suppl_Supplementary_Table_2

vdz029_suppl_Supplementary_Table_3

vdz029_suppl_Supplementary_Table_4

vdz029_suppl_Supplementary_Table_5

vdz029_suppl_Supplementary_Table_6

vdz029_suppl_Supplementary_Table_7

vdz029_suppl_Supplementary_Figure_Table_Legends

## References

[1] Louis DN, Perry A, Reifenberger G, et al The 2016 World Health Organization classification of tumors of the central nervous system: a summary. Acta Neuropathol. 2016;131(6):803–820. 27157931 10.1007/s00401-016-1545-1

[2] Stupp R, Mason WP, van den Bent MJ, et al Radiotherapy plus concomitant and adjuvant temozolomide for glioblastoma. N Engl J Med. 2005;352(10):987–996. 15758009 10.1056/NEJMoa043330

[3] Das S, Marsden PA Angiogenesis in glioblastoma. N Engl J Med. 2013;369(16):1561–1563. 24131182 10.1056/NEJMcibr1309402 PMC5378489

[4] Kotliarova S, Fine HA SnapShot: glioblastoma multiforme. Cancer Cell. 2012;21(5):710–710 e711. 22624719 10.1016/j.ccr.2012.04.031

[5] Keunen O, Johansson M, Oudin A, et al Anti-VEGF treatment reduces blood supply and increases tumor cell invasion in glioblastoma. Proc Natl Acad Sci U S A. 2011;108(9):3749–3754. 21321221 10.1073/pnas.1014480108 PMC3048093

[6] Daubon T, Léon C, Clarke K, et al Deciphering the complex role of thrombospondin-1 in glioblastoma development. Nat Commun. 2019;10(1):1146. 30850588 10.1038/s41467-019-08480-y PMC6408502

[7] Bougnaud S, Golebiewska A, Oudin A, et al Molecular crosstalk between tumour and brain parenchyma instructs histopathological features in glioblastoma. Oncotarget. 2016;7(22):31955–31971. 27049916 10.18632/oncotarget.7454 PMC5077988

[8] Perkins DN, Pappin DJ, Creasy DM, Cottrell JS Probability-based protein identification by searching sequence databases using mass spectrometry data. Electrophoresis. 1999;20(18):3551–3567. 10612281 10.1002/(SICI)1522-2683(19991201)20:18<3551::AID-ELPS3551>3.0.CO;2-2

[9] Benjamini Y, Hochberg Y Controlling the false discovery rate: a practical and powerful approach to multiple testing. Journal of the Royal Statistical Society, Series B. 1995;57(1):289–300.

[10] Kolde R. Pheatmap: pretty heatmaps. R Package Version 61, 1–7 2012.

[11] Soulet F, Kilarski WW, Roux-Dalvai F, et al Mapping the extracellular and membrane proteome associated with the vasculature and the stroma in the embryo. Mol Cell Proteomics. 2013;12(8):2293–2312. 23674615 10.1074/mcp.M112.024075 PMC3734586

[12] Golfinos JG, Norman SA, Coons SW, et al Expression of the genes encoding myelin basic protein and proteolipid protein in human malignant gliomas. Clin Cancer Res. 1997;3(5):799–804. 9815752

[13] Patel AP, Tirosh I, Trombetta JJ, et al Single-cell RNA-seq highlights intratumoral heterogeneity in primary glioblastoma. Science. 2014;344(6190):1396–1401. 24925914 10.1126/science.1254257 PMC4123637

[14] Abe T, La TM, Miyagaki Y, et al Phosphorylation of cortactin by cyclin-dependent kinase 5 modulates actin bundling by the dynamin 1-cortactin ring-like complex and formation of filopodia and lamellipodia in NG108-15 glioma-derived cells. Int J Oncol. 2019;54(2):550–558. 30570111 10.3892/ijo.2018.4663 PMC6317663

[15] Wang J, Miletic H, Sakariassen PØ, et al A reproducible brain tumour model established from human glioblastoma biopsies. BMC Cancer. 2009;9:465. 20040089 10.1186/1471-2407-9-465 PMC2810304

[16] Henriet E, Abou Hammoud A, Dupuy JW, et al Argininosuccinate synthase 1 (ASS1): a marker of unclassified hepatocellular adenoma and high bleeding risk. Hepatology. 2017;66(6):2016–2028. 28646562 10.1002/hep.29336

[17] Ezzoukhry Z, Henriet E, Cordelières FP, et al Combining laser capture microdissection and proteomics reveals an active translation machinery controlling invadosome formation. Nat Commun. 2018;9(1):2031. 29795195 10.1038/s41467-018-04461-9 PMC5966458

[18] Filbin MG, Tirosh I, Hovestadt V, et al Developmental and oncogenic programs in H3K27M gliomas dissected by single-cell RNA-seq. Science. 2018;360(6386):331–335. 29674595 10.1126/science.aao4750 PMC5949869

[19] Kong J, Cooper LA, Wang F, et al Machine-based morphologic analysis of glioblastoma using whole-slide pathology images uncovers clinically relevant molecular correlates. PLoS One. 2013;8(11):e81049. 24236209 10.1371/journal.pone.0081049 PMC3827469

[20] Haferlach T, Kohlmann A, Wieczorek L, et al Clinical utility of microarray-based gene expression profiling in the diagnosis and subclassification of leukemia: report from the International Microarray Innovations in Leukemia Study Group. J Clin Oncol. 2010;28(15):2529–2537. 20406941 10.1200/JCO.2009.23.4732 PMC5569671

[21] Patel VN, Gokulrangan G, Chowdhury SA, et al Network signatures of survival in glioblastoma multiforme. PLoS Comput Biol. 2013;9(9):e1003237. 24068912 10.1371/journal.pcbi.1003237 PMC3777929

[22] Feng H, Liu KW, Guo P, et al Dynamin 2 mediates PDGFRα-SHP-2-promoted glioblastoma growth and invasion. Oncogene. 2012;31(21):2691–2702. 21996738 10.1038/onc.2011.436 PMC3262067

[23] Luwor R, Morokoff AP, Amiridis S, et al Targeting glioma stem cells by functional inhibition of dynamin 2: a Novel treatment strategy for glioblastoma. Cancer Invest. 2019;37(3):144–155. 30907150 10.1080/07357907.2019.1582060

[24] Zorniak M, Clark PA, Leeper HE, et al Differential expression of 2’,3’-cyclic-nucleotide 3’-phosphodiesterase and neural lineage markers correlate with glioblastoma xenograft infiltration and patient survival. Clin Cancer Res. 2012;18(13):3628–3636. 22589395 10.1158/1078-0432.CCR-12-0339 PMC3597469

[25] Rajcevic U, Petersen K, Knol JC, et al iTRAQ-based proteomics profiling reveals increased metabolic activity and cellular cross-talk in angiogenic compared with invasive glioblastoma phenotype. Mol Cell Proteomics. 2009;8(11):2595–2612. 19674965 10.1074/mcp.M900124-MCP200 PMC2773724

[26] Demeure K, Fack F, Duriez E, et al Targeted proteomics to assess the response to anti-angiogenic treatment in human glioblastoma (GBM). Mol Cell Proteomics. 2016;15(2):481–492. 26243272 10.1074/mcp.M115.052423 PMC4739668

[27] Obad N, Espedal H, Jirik R, et al Lack of functional normalisation of tumour vessels following anti-angiogenic therapy in glioblastoma. J Cereb Blood Flow Metab. 2018;38(10):1741–1753. 28627960 10.1177/0271678X17714656 PMC6168744

