# Supplementary figures and images for: The invasive proteome of glioblastoma revealed by laser-capture microdissection
Source: Neurooncol Adv. 2019 Sep 28;1(1):vdz029. doi: 10.1093/noajnl/vdz029 (PMC7212852; doi:10.1093/noajnl/vdz029)

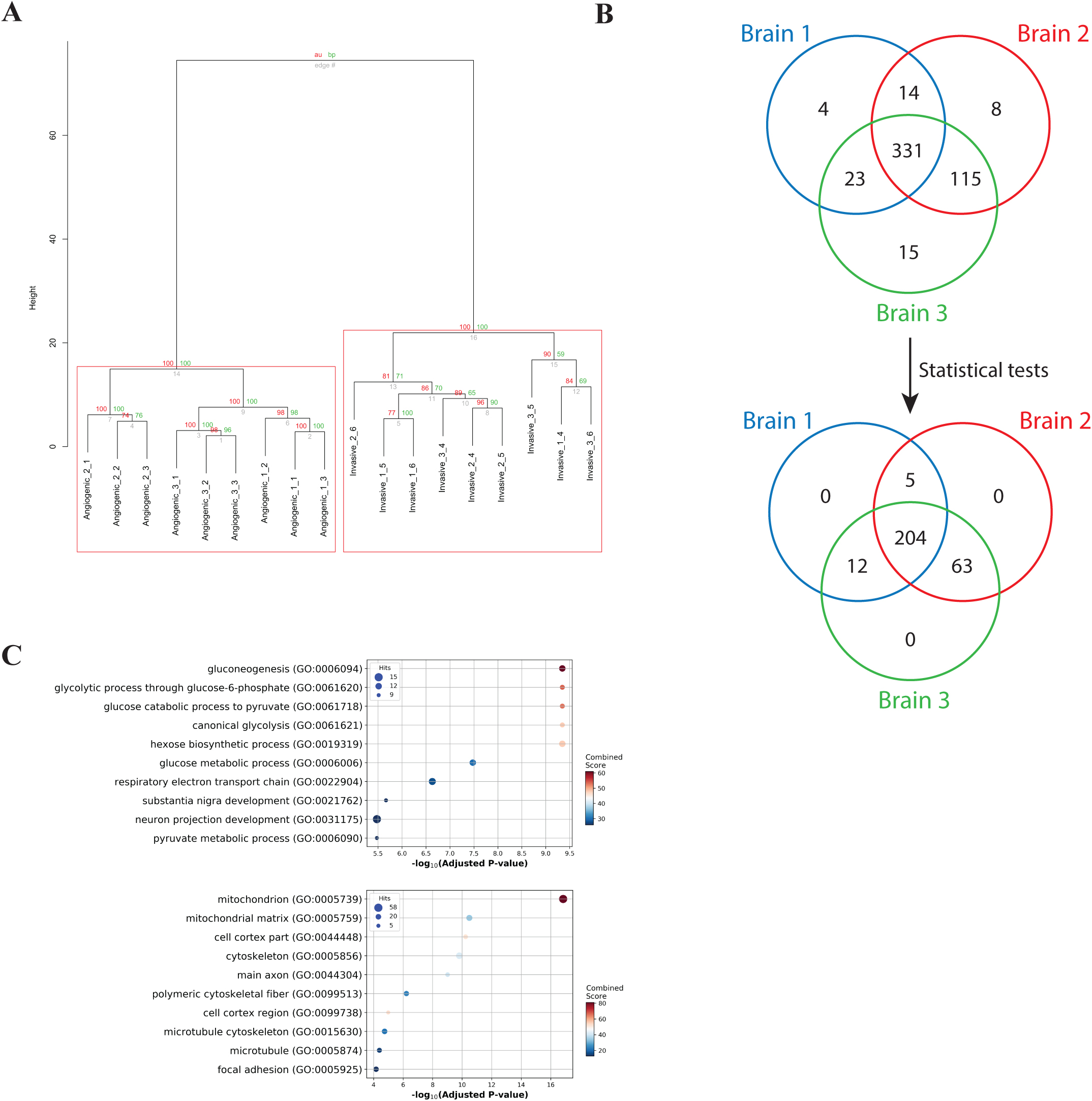

Supplement: vdz029_suppl_Supplementary_Figure_1 [file vdz029_suppl_supplementary_figure_1.jpeg]

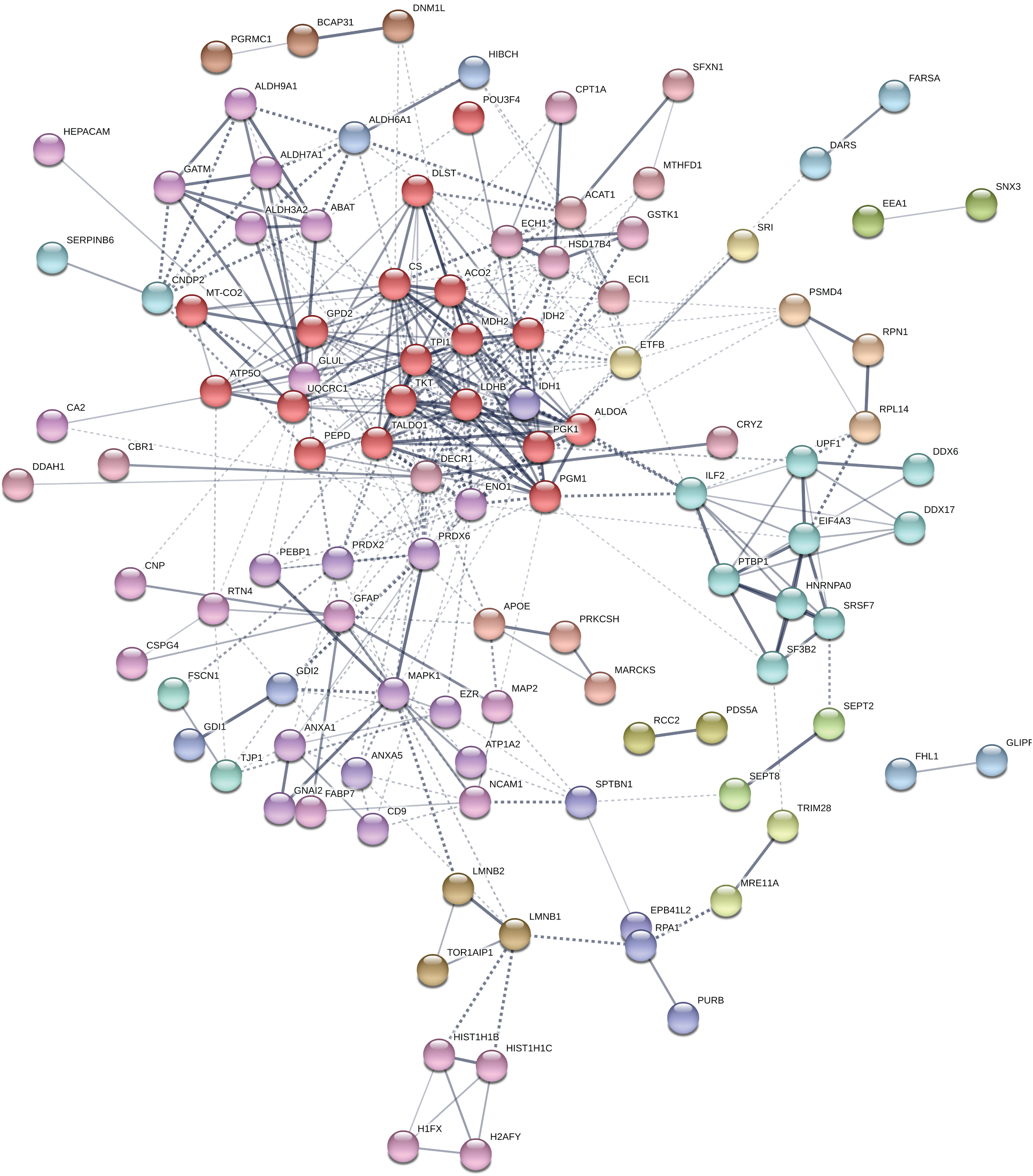

Supplement: vdz029_suppl_Supplementary_Figure_2 [file vdz029_suppl_supplementary_figure_2.jpeg]
