# Supplementary material for: The invasive proteome of glioblastoma revealed by laser-capture microdissection
Source: Neurooncol Adv. 2019 Sep 28;1(1):vdz029. doi: 10.1093/noajnl/vdz029 (PMC7212852; doi:10.1093/noajnl/vdz029)
Supplement: vdz029_suppl_Supplementary_Figure_Table_Legends [file vdz029_suppl_supplementary_figure_table_legends.docx]

**SUPPLEMENTARY INFORMATIONS**

**Supplementary Figure 1: Mouse proteins detected in tumor core and invasive areas**

(**A**) Hierarchical clustering of paired samples obtained from the analysis of the log-ratio values of the 284 mouse proteins common for all samples. Values at the left represent approximately unbiased P values (AU) and values at the right correspond to boot-strap probability (BP).

(**B**) Venn diagrams of mouse proteins expressed in the three different tumors analyzed by proteomics. 331 proteins are common between the 3 tumors (upper diagram). After Welch test p-values by FDR and setting the significance threshold at 0.01, 284 mouse proteins were found differentially regulated between the core and the invasive areas (lower diagram).

(**C**) Gene Set Enrichment Analysis results. Gene Ontology (GO) biological processes (BP) and cellular components (CC) enrichment of the top 284 proteins that are signiﬁcantly differentially expressed between core and invasive areas (pval adj < 0.01 and |logFC| >2), and which are represented here for the three tumor microenvironments. The x-axis represents the negative log10 P-adjusted value. The size of each spot (Ratio) corresponds to the fraction of proteins within our set of proteins that have the corresponding GO function.

**Supplementary Figure 2: Network of core area by using STRING database.**

Each node represent a protein and interactions with medium confidence >0.4 are showed. Proteins are clustered using the Markov Cluster Algorithm and colors represent clusters. Dashed lines represent inter-cluster edges and width represent edge confidence (medium: >0.4, high: >0.7 and highest: 0.9). Disconnected proteins are removed from the analysis.

**SUPPLEMENTARY TABLES**

**Supplementary Table 1**

LC-MS/MS quantitation parameters.

**Supplementary Table 2**

Counts of proteins for Human dataset. For each brain (B1, B2 and B3) we show the number of proteins below the CV threshold for both angiogenic and invasive conditions. Aggregated datasets contain proteins present in at least 6 out of 9 replicates for each condition and common datasets contain proteins present in all replicates of all 3 brain samples.

**Supplementary Table 3**

Counts of proteins for mouse dataset. For each brain (B1, B2 and B3) we show the number of proteins below the CV threshold for both angiogenic and invasive conditions. Aggregated datasets contain proteins present in at least 6 out of 9 replicates for each condition and common datasets contain proteins present in all replicates of all 3 brain samples.

**Supplementary Table 4**

Counts of proteins for Human dataset after filtration.

**Supplementary Table 5**

Counts of proteins for mouse dataset after filtration.

**Supplementary Table 6**

KEGG pathway enriched in invasive areas from Human tumor.

**Supplementary Table 7**

20 best AngioScores. 17 hits with the highest AngioScores on 20 total hits are expressed in the tumor core area.
